# Cellular senescence: Molecular signatures and cellular remodeling

**DOI:** 10.1002/2211-5463.70258

**Published:** 2026-05-05

**Authors:** Dimitris Kletsas

**Affiliations:** ^1^ Laboratory of Cell Proliferation and Ageing, Institute of Biosciences and Applications National Centre of Scientific Research “Demokritos” Agia Paraskevi Athens Greece

## Abstract

Cellular senescence, a state of stable cell‐cycle arrest accompanied by profound metabolic and secretory changes, has emerged as a central hallmark of aging and a key contributor to age‐associated diseases. Despite the great progress in understanding the characteristics, the underlying molecular mechanisms, and the role of senescent cells in several pathologies, many crucial issues remain unresolved. In this ‘In the Limelight’ special issue of *FEBS Open Bio*, three review articles deal with the accurate detection of senescent cells *in vivo*, changes in intercompartmental communication in senescent cells, and the role of lipid metabolism in neuronal senescence.

Cellular senescence is currently recognized as one of the most compelling phenomena in modern biology, with profound implications in tissue homeostasis and age‐related pathologies. First described by Hayflick and Moorehead in the early 1960s [1] as a limit to the serial cultivation of normal cells, overturned the theory of ‘cellular immortality’ proposed by Alexis Carrel from the beginning of the 20^th^ century. They proposed a stable cell‐cycle arrest after repeated subculturing as the core of a normal cell's senescence. This so‐called ‘replicative senescence’ was later linked to telomere attrition, which leads to a DNA damage response and eventually to growth arrest. These cells are characterized by an altered morphology, for example, an enlarged and irregular shape, a lobulated nucleus, and a broad remodeling of cellular compartments and organelles. However, subsequent studies have pointed out a different type of cellular senescence, termed ‘stress‐induced premature senescence’ (SIPS), caused by a variety of genotoxic stresses, including oxidative stress, UV or ionizing radiation, genotoxic drugs, inflammatory compounds, and even the activation of certain oncogenes [2]. The inability of these cells to proliferate, and thereby inhibit malignant transformation, defines the so‐called ‘bright side of cellular senescence’, representing a powerful tumor‐suppressive mechanism.

Although senescence has been described since the 1960s, it was regarded by many researchers as a laboratory artifact caused by culture conditions rather than a real biological phenomenon happening *in vivo*. It was only in the mid‐1990s that the presence of senescent cells in tissues of aged individuals or in pathologic states was demonstrated using specific markers [3]. This observation prompted senescence to become a hot topic in modern biology. Consequently, a series of studies have provided extensive evidence indicating other hallmarks of senescence beyond a stable cell‐cycle arrest, that is, macromolecular damage, an altered metabolism and a senescence‐associated secretory phenotype (SASP) [2]. SASP represents a pro‐inflammatory and catabolic phenotype—characteristic of the cell type and the senescence stimulus—that can locally affect tissue homeostasis. A series of evidence indicated that the accumulation of senescent cells in the tissues can reshape the tissue microenvironment and contribute to age‐related pathologies, including vascular disorders, obesity, fibrotic diseases, type 2 diabetes, renal diseases, musculoskeletal disorders, sarcopenia, and cancer [4], collectively describing the ‘dark side of senescence’. Concerning cancer, although senescence, as mentioned above, represents a potent barrier against malignant transformation (alongside with apoptosis and immune surveillance), the accumulation of senescent cells in the tissues, due to SASP, can create a ‘permissive environment’ that enhances tumor growth, portraying senescence as a ‘double‐edged sword’ in cancer development [5].

According to the above, cellular senescence represents a novel target of aging and age‐related diseases [6]. A series of compounds that reduce the load of senescent cells by suppressing or inhibiting SASP, termed senomorphics, have been identified. In addition, senolytics are compounds that selectively kill senescent cells based on the resistance of these cells to certain apoptotic stimuli. This general class of senotherapeutics may prove to be a major anti‐aging arsenal, especially when problems of tissue specificity and side effects are overcome. In this ‘In the Limelight’ special issue of *FEBS Open Bio*, three review articles that address major topics and recent developments in the field are presented.

As mentioned above, the identification of specific biomarkers for cellular senescence is crucial for their accurate detection in tissues *in vivo*. Over the years, several markers have been proposed based on morphological traits or molecular features that also characterize other cellular processes not necessarily linked to senescence. Thus, by overlooking the heterogeneity and complexity of the senescence process, these markers fail to serve as specific indicators. In this issue, Ntintas *et al*. address this point [7]. Their group recently presented lipofuscin accumulation as a hallmark of senescent cells [8], which was also incorporated into a guideline algorithm for the accurate detection of senescence [2]. In their review, they present an analysis of several senescence signatures developed by different groups. To this end, they used several predefined criteria (i.e., sensitivity, specificity, cellular diversity, tissue coverage, cross‐species applicability, and high‐throughput data validation) and discussed the strengths and limitations of these approaches, concluding that there is a need for a signature that addresses all the above‐mentioned criteria.

Senescent cells are characterized by significant alterations in morphology, the most common being the increased size, the loss of typical morphology (e.g., the loss of spindle shape in fibroblasts), and the enlargement and lobulation of the nucleus. On other hand, proper cell compartmentalization and intercompartmental communication are essential for responding to stimuli and for the transfer of information and materials, thereby ensuring cell function. In this issue, Mazan‐Mamczarz *et al*. [9] describe the extensive remodeling of membrane‐bound cellular compartments in senescent cells, including the plasma membrane, nucleus, endoplasmic reticulum, Golgi, mitochondria, and the cytoskeleton. They further discuss how these disturbances disrupt the appropriate coordination of cellular processes and the proper transfer of information with broad consequences for the function of senescent cells.

Metabolic reprogramming is a hallmark of cellular senescence [2]. Previous studies have investigated lipid metabolism in classical models of senescence, such as in fibroblasts, but recently this research has been extended to neuronal and non‐neuronal cells in the brain. In this issue, Tsagkari *et al*. [10] describe alterations in lipid synthesis, transport, and storage in different cell types in the brain, for example, neurons, astrocytes, microglia, and other glial cells, in a cell type‐specific manner. They also provide evidence to show that imbalances in different lipid species can act as drivers or consequences in brain aging and in neurodegenerative diseases. Finally, they discuss whether interventions with senotherapeutics or lipid regulating drugs hold promise for treating senescence‐related neurodegenerative disorders.

I would like to express my gratitude to all authors for their inspiring contributions to this special ‘In the Limelight’ issue of *FEBS Open Bio*, which highlights crucial topics in cellular senescence research.

## Conflict of interest

The author declares no conflict of interest.

## Author contributions

DK wrote the editorial.
